# Dynamical EEG Indices of Progressive Motor Inhibition and Error-Monitoring

**DOI:** 10.3390/brainsci11040478

**Published:** 2021-04-09

**Authors:** Trung Van Nguyen, Prasad Balachandran, Neil G. Muggleton, Wei-Kuang Liang, Chi-Hung Juan

**Affiliations:** 1 Institute of Cognitive Neuroscience, National Central University, Taoyuan City 32001, Taiwan; trungnv1087@gmail.com (T.V.N.); prasad.aquarius93@gmail.com (P.B.); neil.muggleton@gmail.com (N.G.M.); weikuangliang@gmail.com (W.-K.L.); 2 Taiwan International Graduate Program in Interdisciplinary Neuroscience, National Cheng Kung University and Academia Sinica, Taipei 11529, Taiwan; 3 Cognitive Intelligence and Precision Healthcare Center, National Central University, Taoyuan City 32001, Taiwan; 4 Department of Psychology, Kaohsiung Medical University, Kaohsiung City 80708, Taiwan

**Keywords:** inhibitory control, force, partial error, error correction, error monitoring, selective stop signal task, Hilbert–Huang transform

## Abstract

Response inhibition has been widely explored using the stop signal paradigm in the laboratory setting. However, the mechanism that demarcates attentional capture from the motor inhibition process is still unclear. Error monitoring is also involved in the stop signal task. Error responses that do not complete, i.e., partial errors, may require different error monitoring mechanisms relative to an overt error. Thus, in this study, we included a “continue go” (Cont_Go) condition to the stop signal task to investigate the inhibitory control process. To establish the finer difference in error processing (partial vs. full unsuccessful stop (USST)), a grip-force device was used in tandem with electroencephalographic (EEG), and the time-frequency characteristics were computed with Hilbert–Huang transform (HHT). Relative to Cont_Go, HHT results reveal (1) an increased beta and low gamma power for successful stop trials, indicating an electrophysiological index of inhibitory control, (2) an enhanced theta and alpha power for full USST trials that may mirror error processing. Additionally, the higher theta and alpha power observed in partial over full USST trials around 100 ms before the response onset, indicating the early detection of error and the corresponding correction process. Together, this study extends our understanding of the finer motor inhibition control and its dynamic electrophysiological mechanisms.

## 1. Introduction

The ability to suppress a pre-potent motor response to adjust to rapid environmental changes is a major executive control function. Impairment in motor inhibitory control is linked to numerous clinical ailments including attention deficit hyperactivity [1], obsessive-compulsive disorder [2], Tourette’s syndrome in adults [3] and Parkinson’s disease with surgical treatments such as subthalamotomy or deep brain stimulation [4,5] and without those special treatments [6,7]. This process has been widely studied both in a clinical population and healthy volunteers using the stop signal task [8,9]. In each trial, participants are required to respond as quickly as possible to an imperative stimulus, but are instructed to abort their response if the imperative stimulus was subsequently followed by a stop signal, which typically has a variable onset time with respect to the imperative stimulus. The execution of inhibitory control is described using the independent horse race model [8,10,11] with the race being between a “go” process for a response and a “stop” process when a stop signal is presented. Neurophysiological studies have illustrated that the neural correlates of the go and stop processes produce saccadic eye movements through an interactive race model [12]. In the independent horse race model, go and stop processes are autonomous of each other and the successful or unsuccessful inhibition of the response depends on which of the two processes finishes first. The latency of the stop process, referred to as the stop signal reaction time (SSRT), can be estimated from the observed probability of stopping and the reaction time on go trials [8,9,13].

In addition to the inhibition process, the stop signal task also involves error monitoring, a process that plays an important role in executive functions. Alteration of the time delay (stop signal delay; SSD) between the go and the stop signal based on task performance accuracy of an individual can be used to ensure that the participants will fail to inhibit (unsuccessful stop, USST) their responses on around 50% of trials. The failure to inhibit a response results in pressing a key (overt error) on a trial where the key press should be withheld. The error response leads to behavioral change, which is seen in modulation of reaction times and accuracy on subsequent trials [14,15,16]. In particular, error monitoring refers to error detection and response adjustments after errors [17] and specific electrophysiological correlates accompany these modulations. Electroencephalographic (EEG) studies show that power in theta (4–7 Hz) and alpha (8–13 Hz) bands increase over the frontal-central region as a neural marker of the error monitoring [18,19,20,21]. Theta band power is seen to be enhanced in the medial frontal cortex after error detection [22]. Theta oscillations after error response are observed to have a positive correlation with both post-error accuracy [23] and post-error slowing of responses [24]. In addition to overt errors, partial errors have also been observed in the responses by using force measurement or recording the EMG activity [25,26,27]. The occurrence of partial responses shows that participants are likely to also detect, inhibit and correct errors prior to producing full responses [28,29]. However, the dynamics of error-related neuro-markers in time-frequency domains that occur during partial error still remains unknown.

To identify the electrophysiological markers for response inhibition, most previous studies have applied time-frequency analyses to extract the frequencies that are maximally associated with the response inhibition process. Practically, prior findings have suggested a functional role for beta oscillation in motor inhibition [30,31]. For example, a larger beta power was observed for successful stop trials (SST) than for USST in 6-year-old children [31]. Swann et al. [32] also found stronger beta power in SST in an ECoG study than in USST at the right inferior frontal gyrus (rIFG). In another ECoG study, a strengthened beta oscillation was observed in the rIFG and pre-SMA associated with successful response inhibition [33]. Moreover, strengthened beta power was observed when monkeys made a response inhibition decision in a Go/NoGo task [34]. These findings suggest that beta power may be a critical indicator of response inhibition. Most previous studies have investigated response inhibition using the stop signal task. To identify the inhibitory processes, SST trials were contrasted with USST. This comparison of SST with USST trials may largely relate to error processing [35]. Alternatively, SST trials were contrasted with go trials to evaluate inhibitory processes. However, an abruptly appearing stop signal in the classical stop signal task is itself likely to involve both attentional capture (i.e., the onset of an extra signal compared to go trials) and response inhibition processes simultaneously [36,37]. Therefore, in this version of the task, inhibitory processing is likely occurring alongside attention capture meaning that the oscillatory EEG processes are still equivocal and require further investigation [38].

Our previous study employed a selective stop signal task, using continue go trials (Cont_Go) as the baseline for stop trials in order to control for the attentional capture in stop signal trials (Nguyen et al. [27]; see also Sharp et al. [37]). In these trials, an unexpected signal emerges to instruct subjects to sustain their go response in order to imitate the attentional capture effect in the stop trials. Moreover, force measurement was used to capture partial and full USST trials that are relevant to error detection and error correction processes. In the current study, we relied on the same measure as in our prior study (Nguyen et al. [27]) to explore the electrophysiological mechanisms that might be related to inhibition, error detection and behavior correction. There were three primary aims of this study: (1) to determine inhibition-related brain dynamics; (2) to determine error-related brain dynamics; (3) to investigate whether the error correction processes can be observed in the spectral characteristics of EEG signal in the immediately following erroneous responses—i.e., on trials with “partial responses”. To answer these questions, we compared SST vs. Cont_Go as well as full USST vs. Cont_Go to explore the neural mechanism of inhibition and error detection processing, respectively. We also compared partial vs. full USST to identify the neural mechanism of error correction processing.

## 2. Materials and Methods

The current study utilized the same data samples from the participants in our prior publication (Nguyen et al. [27]). The participant data were subjected to novel analysis methods to account for the dynamic changes in the brain by assessing their neural oscillations, i.e., EEG rather than Event related mode/Event-related potentials (ERM/ERPs) (as utilized in our prior publication). Additionally, by employing novel analysis methods on the same participant data, the current study was able to investigate research questions and validate relevant hypotheses different from our prior publication. In the following section, we briefly describe the key features of the task; further details can be found in [27].

### 2.1. Participants

Twenty healthy right-handed undergraduate and graduate students with normal or corrected-to-normal visual acuity were recruited from National Central University, Taiwan. This study was approved by the Institutional Review Board of the Chang Gung Memorial Hospital, Linkou, Taiwan (Ethical Approval Code is 201601724A3C103). Written informed consent was attained from all participants prior to their participation.

### 2.2. Selective Stop Signal Task

**Figure 1:**
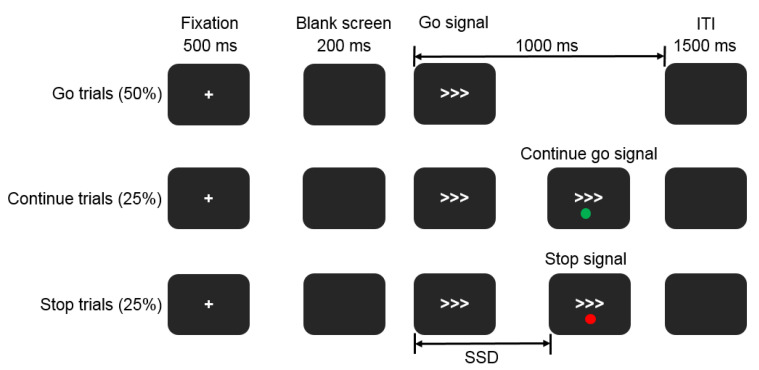
Selective stop signal paradigm. Red and green dots representing stop or continue go trials were displayed either above or below the go signal with their color/instruction offset across participants. Note: SSD: stop signal delay; ITI: inter-trial interval.

Three trial types were conducted in the selective stop signal task: go, stop and continue go (Cont_Go) trials (Figure 1). A go trial commenced with a 500 ms central fixation of a white cross and a subsequent 200 ms blank screen. Next, the go signal (a right or left-pointing arrow) was presented for 1000 ms and was followed by a blank screen with an inter-trial interval (ITI) of 1500 ms. Participants responded using a force pincher in their left (right) hand according to the left (right) pointing arrow. In a random sub-set of trials, a dot was shown above or below the go stimulus, signifying that participants were to suppress their responses. In the Cont_Go trials, a different colored dot was used as a signal, also emerging above or below the go signal. For these trials, participants were required to not modify their actions and to continue to respond to the go stimulus. The dots representing stop and Cont_Go trials were red and green and were balanced across subjects such that half had red for stop and half had green for stop. A total of 10 blocks of 64 trials each were displayed. The likelihood of a stop signal and a Cont_Go signal emerging on trial were equivalent, both with a 25% chance (thus, trials were 50% go, 25% stop and 25% Cont_Go). The three types were pseudo-randomized to prevent more than either two stop trials or two Cont_Go trials being presented in a row. The leftward and rightward go stimuli were equally frequent.

### 2.3. Electroencephalography Recording

Electroencephalography data were collected at a sampling rate of 1 kHz from each of 36 electrodes distributed over both hemispheres in a 10/20 system arrangement. The ground electrode was positioned over Fz. Each channel’s signal was referenced online to the average of electrodes at the left and right mastoids (A1 and A2). The vertical and horizontal electrooculograms (EOG) were also collected. The impedance of all EEG electrodes was maintained below 10 kΩ, and the EEG data were re-referenced offline to the whole-head average.

### 2.4. Data Analysis

#### 2.4.1. Behavioral Analysis

In current study, USST trials were further divided into partial and full USST trials following the distribution of the go response peak force values. Firstly, the mean and standard deviation of the peak force of go were calculated. Should the peak force of USST be smaller than the (M—3*SD) force of go, then it was scored as a partial USST; otherwise, it was a full USST.

#### 2.4.2. EEG Data

##### Hilbert–Huang Transform (HHT)

The continuous EEG data were epoched into 1800 ms epochs from −800 ms to 1000 ms relative to stop/Cont_Go stimulus onset (hereafter referred to simply as “stop onset” for convenience). The epoch lengths selected were long enough to exclude the possibility of noise/edge effects in the analysis. Ocular artifacts (blink or saccades) were isolated and removed by an independent component algorithm. Epochs were followed by artifact rejection with a ±100 μV threshold criteria for each channel [39]. Following artifact rejection, the EEG data of all trials in individual channels were standardized by dividing by their standard deviation, producing a unit-free measure of amplitude. The HHT analysis was applied to extract time-frequency data from the EEG signals.

The HHT comprises empirical mode decomposition (EMD) and Hilbert spectral analysis transform [40,41]. The EMD is fully adaptive and data-driven to decompose a signal like EEG into numerous intrinsic mode functions (IMFs). These IMFs characterize different oscillatory modes within the data. To determine the mode-mixing problem that the original EMD method might cause, the current study functionalized ensemble EMD (EEMD) [42], a noise-assisted version of EMD, for each trial. To perform EEMD, the IMFs were generated from the collective means of trials by repeating the original EMD process on the same signal using diverse sets of Gaussian noise. The current EEMD analysis was applied with 100 ensembles and a noise level of 0.2. Subsequently, the Hilbert spectrum was calculated for each trial and each IMF to obtain the instantaneous frequencies and amplitudes. The HHT was executed with customized MATLAB (MathWorks) scripts with EEMD code supplemented by the Research Center for Adaptive Data Analysis of National Central University, Taiwan. Additional data processing and statistical analysis were achieved using SPM8 for Magnetoencephalography (MEG) and EEG (Wellcome Department of Cognitive Neurology, London, United Kingdom).

The averaged oscillatory power for each condition for each participant was rescaled by subtracting the mean signal from −300 to 0 ms relative to the stop signal onset. For statistical analyses, a two-tailed cluster-based nonparametric permutation (CBnPP) test [43,44,45] was utilized to assess the alterations of the multichannel HHT spectra (channels × frequency × time points) between each of paired conditions at the sensor level. Initially, this technique was applied to offer weak family-wise error rate (FWER) control for EEG/MEG data by assembling assessment outcomes at neighboring sensors and time points into clusters based on their statistical significance and proximity. If the distance between two sensors was less than 60 mm, they were recognized as neighbors in the current study. For all statistical tests, 5000 permutations were performed. CBnPP is a powerful method to reveal substantial effects, especially clustered effects, in EEG data. Compared with Bonferroni or false discovery rate correction, CBnPP is a less conservative approach for multiple comparison errors.

## 3. Results

### 3.1. Behavior Results

All behavior results were as reported in Nguyen et al. [27]. Briefly, the mean of stop signal reaction time was 219.2 ms (SD = 31 ms). The number of partial USST trials for participants ranged from 8 to 78 (M = 35.6, SD = 17.6) and constituted 46.5% (SD = 13.9) of USST trials, while the numbers of full USST trials for participants ranged from 25 to 51 (M = 37.8, SD = 8.3) and constituted 53.4% (SD = 13.9) of USST trials. The peak force of go and Cont_Go were significantly greater than in both partial and full USST (*p* < 0.01). Moreover, a significantly larger peak force was evident for full USST trials compared to partial USST trials (*p* < 0.01). The RT of partial USST was significantly longer compared to both the RT of full USST and the RT of go trials (*p* < 0.05), while the RT of full USST was significantly faster than the RT of go trials (*p* < 0.05).

### 3.2. HHT Results

#### 3.2.1. Inhibitory Control

**Figure 2:**
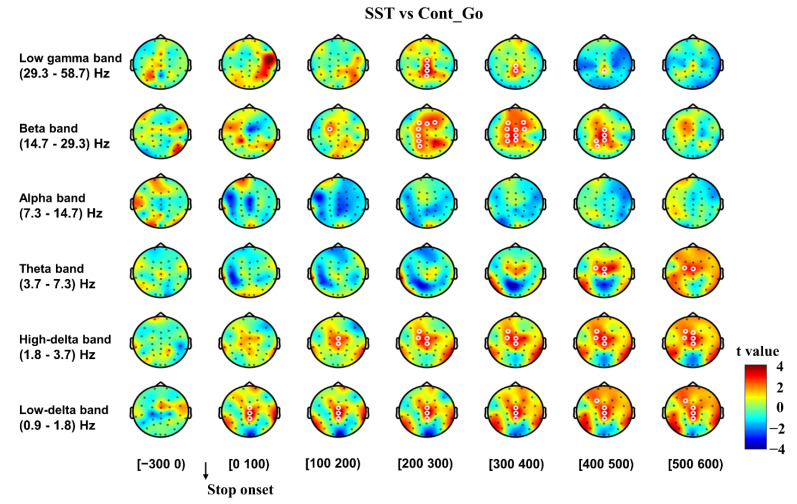
Topography of the differences in time-frequency spectrum between successful stop trials (SST) and “continue go” (Cont_Go) trials in the time window (−300 to 600 ms). The time window was separated into seven-time bins with each topography indicating a one-time bin. The time window of the baseline consisted of a single time bin of 300 ms. Data for analysis from all trials were time-locked to the stop onset. Note: The color shade illustrates the t value (red color denotes positive t value, and blue denotes negative t value). White circles around an EEG channel indicate a significant effect for that channel, *p* < 0.05, two-tailed CBnPP test.

To analyze the neural oscillatory dynamics of inhibitory control, data from all trials were time-locked to the stop onset, and the contrast of SST and Cont_Go trials was performed (Figure 2). The delta band power (0.9–3.7 Hz) significantly increased in the SST compared to the Cont_Go condition (*p* < 0.05, CBnPP) in the 0–600 ms time window after the stop signal onset. Significantly increased theta band power was observed for the SST condition compared to the Cont_Go condition from 400 ms after stop onset until 600 ms for central areas (*p* < 0.05, CBnPP). However, no significant difference in alpha oscillations (*p* > 0.05, CBnPP) was detected between SST and Cont_Go conditions. Significantly higher beta power was observed for the SST condition than for the Cont_Go condition from 100 ms after stop onset until 500 ms in the frontal, central and parietal areas (*p* < 0.05, CBnPP). Finally, the low gamma band power was significantly higher in the SST than in the Cont_Go from 200 ms after stop onset until 500 ms in central and parietal areas (*p* < 0.05, CBnPP). The increase in beta and low gamma activity was observed specifically after a stop signal and before the end of the SSRT (219 ms).

**Figure 3:**
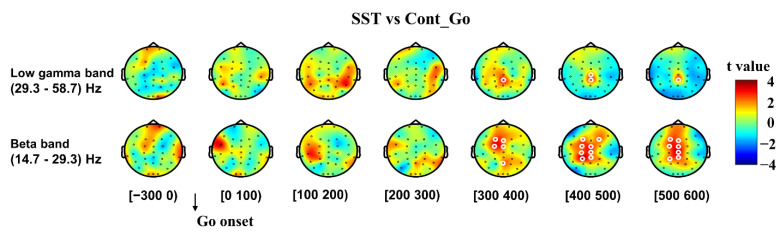
The topography of the differences in time-frequency spectra between SST and Cont_Go in the time window (−300 to 600 ms). The time window was separated into seven-time bins with each topography indicating a one-time bin. Data for analysis from all trials were time-locked to the go signal onset. Note: The color shading indicates the t value (red color denotes a positive t value, and blue denotes a negative t value). White circles around an EEG channel indicate a significant effect for that channel, *p* < 0.05, two-tailed CBnPP test.

Data from SST and Cont_Go trials were also time-locked to the go onset, and comparisons of SST and Cont_Go trials were performed to analyze neural oscillatory dynamics of inhibitory control (Figure 3). Similar to the increase in power observed for the stop-locked analysis (Figure 2), significantly increased beta power was also evident in the SST compared to the Cont_Go condition from 300 ms after go onset until 600 ms in frontal, central and parietal areas (*p* < 0.05, CBnPP). The low gamma band’s power was significantly higher in the SST than in the Cont_Go (*p* < 0.05, CBnPP).

**Figure 4:**
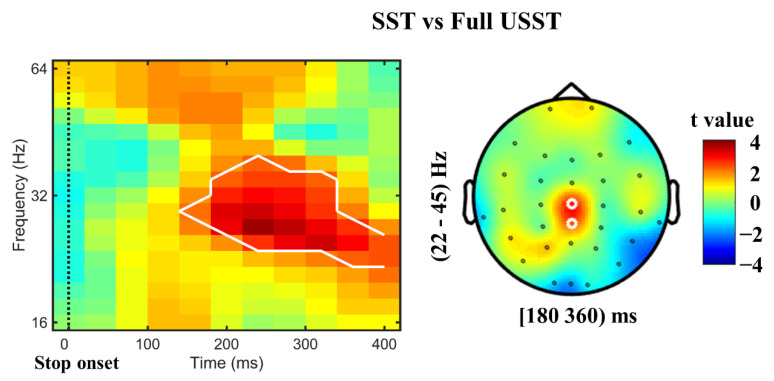
Contrast of beta and gamma power between SST and full USST. Left panel: stop-locked time-frequency power over Cz electrodes for SST minus full USST condition. Right panel: the topography for SST minus full USST trials. The significance for this difference was observed at Cz and CPz electrodes. Note: The color shading indicated the t value (red color denotes a positive t value, and blue denotes a negative t value). White circles around an EEG channels indicate a significant effect for that channel, *p* < 0.05, two-tailed CBnPP test.

To reproduce the results of preceding studies [31,32], a comparison of SST and full USST was also performed. The power in beta and low gamma band (22–45 Hz) in SST was significantly higher than in full USST from 180 to 360 ms after stop onset (*p* < 0.05, CBnPP; left Figure 4). The right-hand side Figure 4 shows the topography of the comparison. Significantly higher beta and low gamma power (22–45 Hz) was seen for the SST than for the full USST condition in central and parietal areas (Cz and CPz).

#### 3.2.2. Error Detection and Error Correction

**Figure 5:**
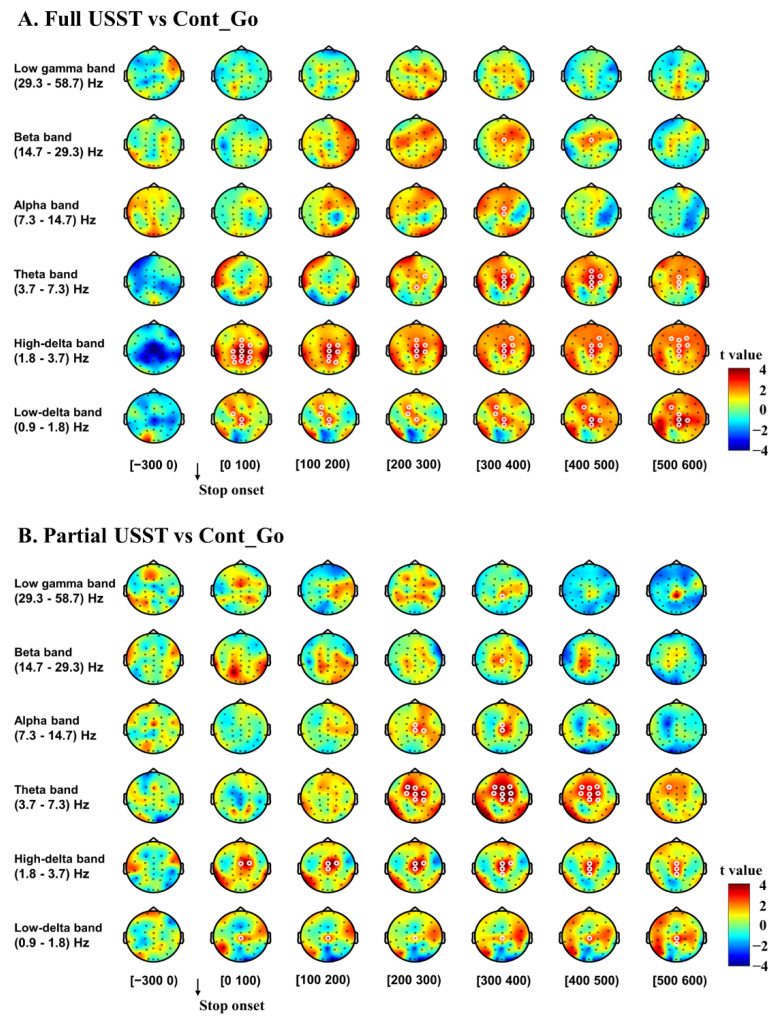
Topography of the differences in time-frequency spectrum between full USST and Cont_Go (**A**) as well as for partial USST and Cont_Go (**B**) in the time window from −300 to 600 ms. The trial time window was separated into seven-time bins with each topography shown for each time bin. The time window of the baseline consisted of a single time bin of 300 ms. Data for analysis from all trials were time-locked to the response onset. Note: the right color bar displays the t value (red denotes positive t values, and blue denotes negative t values). White circles around an EEG channel indicate a significant effect for that channel, *p* < 0.05, two-tailed CBnPP test.

To help understand the mechanisms of error detection and error correction, data from USST (full and partial) and Cont_Go trials were time-locked to the stop onset, and the contrast between paired conditions was performed (Figure 5). The power in the delta band was significantly higher in the full USST compared to the Cont_Go condition (*p* < 0.05, CBnPP) in the 0–600 ms time window after the stop onset in the frontal, central and parietal areas (Figure 5A). Significantly increased theta power was observed in the full USST compared to the Cont_Go condition from 200 ms after stop onset until 600 ms in the central area (*p* < 0.05, CBnPP). Moreover, significantly higher alpha power was also seen for the full USST condition relative to the Cont_Go condition from 300 ms after stop onset until 400 ms at central areas (*p* < 0.05, CBnPP). Significantly increased beta power was also seen for the full USST compared to the Cont_Go condition from 300 ms after stop onset until 500 ms in central areas (*p* < 0.05 CBnPP). However, there was no significant difference in low gamma oscillations (*p* > 0.05, CBnPP) between SST and Cont_Go conditions.

Figure 5B shows the results of the comparison between partial USST and Cont_Go. The delta band power was higher in the partial USST than in the Cont_Go condition (*p* < 0.05, CBnPP) for the 0–600 ms time window after stop onset. Significantly increased theta power was also evident in the partial USST compared to the Cont_Go condition from 200 ms after stop onset until 600 ms (*p* < 0.05 CBnPP) at frontal and central areas. Moreover, significantly increased alpha power was also evident in the partial USST compared to the Cont_Go condition from 400 ms after stop onset until 600 ms at central expanses (*p* < 0.05, CBnPP). Significantly higher beta and low gamma power were also seen for the partial USST than for the Cont_Go condition from 300 ms after stop onset until 400 ms at central areas (*p* < 0.05, CBnPP). 

**Figure 6:**
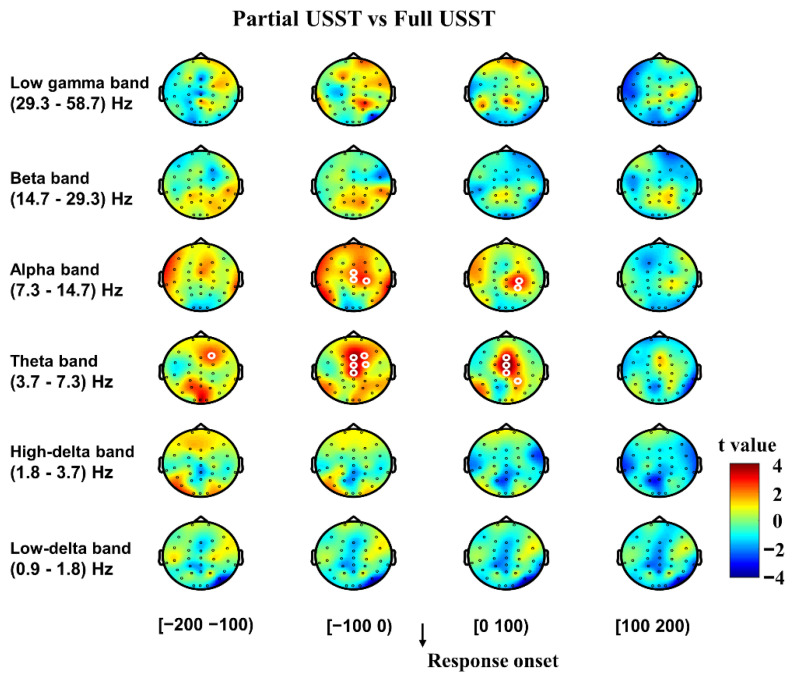
The topography of the difference in time-frequency spectra between partial USST and full USST in the time windows from −200 to 200 ms. The time window was separated into four-time bins with each topography indicating one-time bin (100 ms). Data for analysis from all trials were time-locked to the response onset. Note: The right color bar displays the t value with red for positive t values and blue for negative t values. White circles around an EEG channel indicate a significant effect for that channel, *p* < 0.05, two-tailed CBnPP test.

**Figure 7:**
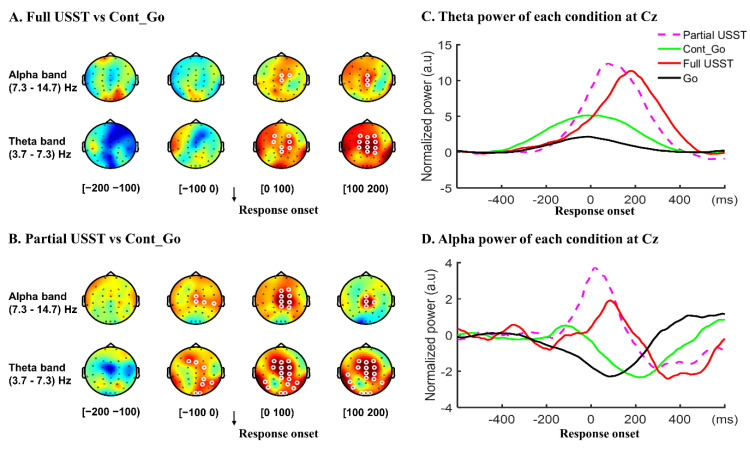
Theta and alpha power of each condition. The topography of theta and alpha power of the difference between full USST and Cont_Go (**A**) as well as for partial USST and Cont_Go (**B**) in the time windows from −200 to 200 ms. White circles around an EEG channel indicate a significant effect for that channel, *p* < 0.05, two-tailed CBnPP test. (**C**): mean theta power of each condition at Cz. (**D**): mean alpha power of each condition at Cz.

Following these outcomes, we further analyzed the response-locked power spectrum fluctuations to differentiate the neural mechanisms between partial and full USST conditions. All trials were time-locked to the response onset, and the contrast between partial and full USST was also performed (Figure 6). There was no significant difference in the delta, beta and gamma oscillations between partial and full USST conditions (all *p* > 0.05, CBnPP). However, theta and alpha band powers were significantly higher in the partial USST than in the full USST condition (*p* < 0.05, CBnPP) from 100 ms before response onset until 100 ms after response onset. Likewise, Figure 7 shows that theta and alpha power of partial USST increase earlier than in full USST in the 100 ms before response onset when Cont_Go was used as baseline. Theta and alpha band power was higher in the full USST than in the Cont_Go condition from 0–100 ms after response onset (*p* < 0.05, CBnPP) (Figure 7A), whereas those bands’ power was higher in partial USST than in the Cont_Go condition from 100 ms before response onset until 100 ms after response onset (Figure 7B). Moreover, upon visual inspection, the peak in theta and alpha was much earlier in partial USST compared to full USST (Figure 7C,D).

## 4. Discussion

The aim of the current study was to explore the neural mechanisms of response inhibition and error monitoring through force measurement and EEG indices for specific frequency band activities. The results demonstrate that a larger beta and low gamma power was observed in SST trials compared to Cont_Go trials, showing that beta and low gamma band is associated with the inhibition process [31,32]. There was no difference in the alpha band between SST and Cont_Go, suggesting that the Cont_Go and stop signal evoked the same capture attention level. Furthermore, higher alpha activity and theta activity after the response onset was observed in full USST trials compared to Cont_Go trials, indicative of error detection processing [18,46]. Interestingly, higher alpha and theta activity was observed in partial over full unsuccessful stop trials and Cont_Go in the time window from 100 ms prior to response onset until response onset. These results suggest that the increased theta and alpha in partial USST is associated with early detection of errors and the corresponding correction process. To our knowledge, this is the first study that reports EEG evidence for theta and alpha activity relating to error correction processing.

### 4.1. Neural Mechanisms of Motor Inhibitory Control

The increased beta band activity for SST related to full USST was consistent with previous studies [31,32,33] that validated the phenomena of inhibition in the presented task. Some studies have shown that contrasting SST with USST trials indicates the involvement of error processing [35]. Therefore, the functional role of beta activity remains unclear as to whether it is a consequence of inhibition or error processing. In the current study, the Cont_Go condition was used as the baseline in comparison with the SST condition instead of full USST to control for capture of attention from the stop signal and evaluate possible error processing during the wrong response. The current time-frequency results indicate that beta band activity increased for SST related to Cont_Go. A possible explanation for this might be that the beta band activity is related to maintaining the current brain state or that it “signals the status quo” [47]. In contrast, beta frequency EEG responses are reduced in amplitude during preparation for a movement and increase in amplitude when the movement ends (beta rebound). Therefore, the larger beta power activity observed in the stop condition relative to the Cont_Go condition may arise from the no response after the stop signal presentation. In other words, the beta band activity in SST trials was less desynchronized than in Cont_Go trials that consolidates the change from motor rest condition to response production. Moreover, Gilbertson and colleagues showed that the increased beta band activity is accompanied with diminished motor performance [48]. In another study, applying transcranial alternating current stimulation over M1 resulted in decreased finger movement velocity [49] and force [50]. These patterns of results suggest that the enhanced beta band reflects the motor inhibition process.

On the other hand, many priori studies have showed that the inhibitory processes are associated with several cortical and subcortical structures. A number of neuroimaging studies have suggested that the pre-supplementary motor area (pre-SMA) and the striatum are more involved in inhibitory control [51,52,53,54,55]. The right inferior frontal gyrus (rIFG) is also involved in inhibitory control [56,57]. However, its role is still debated. Recent studies have reported that rIFG reflects attentional capture rather than response inhibition [37,58]. In ECoG studies, beta oscillations in pre-SMA, rIFC and subthalamic nucleus (STN) are increased when a movement is successfully inhibited [59,60]. Moreover, several studies have shown that bilateral STN deep brain stimulation actually leads to improved response inhibition [61,62,63,64]. Finally, M1 and premotor dorsal cortex have been well established to be actively involved with inhibitory control both during action planning and its inhibition in monkey [65,66] and in human [67] studies. The intracranial electrode ERP results from epileptic patients during pre-surgical monitoring showed that stop ERPs occur mainly in M1 and the premotor dorsal cortex when patients successfully stopped their movement [68]. The results suggest a causal involvement of the motor cortices in inhibitory control. Interestingly, such ERPs were observed for a fraction of the wrong strop trials, i.e., when an individual moved their arm instead of cancelling the movement. Longer RTs accompanied with these wrong trials suggest a possible late modulation of the neural activity, incapable of suppressing an arm movement. In the current study, we found that the neural activity in frontal and central regions related to response inhibition was in agreement with neuroimaging (the medial prefrontal areas [54,55]) and ECoG studies (M1, dorsal premotor cortex [67,68]). Together, these findings suggest that beta band activity may serve as a potential electrophysiological index of inhibitory control when additional cognitive functions such as capture attention or error processing are controlled.

In addition to the beta band patterns, stronger central low gamma power was also observed in SST condition compared to the Cont_Go condition in central areas. This increase was observed specifically after a stop signal and before the end of the SSRT. These results suggest an association of low gamma oscillations with inhibitory control and also agree with prior evidence [31,69] that low gamma may be associated with the inhibition process. Shibata et al. [69] and Lo et al. [31] employed different tasks such as Go/NoGo and stop signal task, respectively, to look at inhibitory control. Shibata and co-workers observed an increase in low gamma band (31 Hz) activity in the central region in the NoGo condition of their task, suggesting that the low gamma band oscillations may be associated with the inhibition process [69]. Lo and colleagues reported a relationship between low gamma band and the ability to inhibit motor responses with increased frontal low gamma band oscillations in the SST coupled with quicker SSRTs [31]. In the current study, movement occurred during Cont_Go trials, while none occurred during SST trials, and it was expected that low gamma activity would be smaller in the former. Therefore, an increase in low gamma band activity in successful stop trials relative to Cont_Go suggests an association of low gamma oscillations with inhibitory control.

A larger delta power was observed for the SST trials compared to the Cont_Go trials. This result is in line with prior studies [38,70] that suggest that delta oscillations may serve as a potential electrophysiological index of motor inhibitory control. We also observed significantly larger delta power for the USST trials (partial USST and full USST) compared to the Cont_Go trials, while no significant difference was observed between full USST and partial USST. Moreover, comparing the delta power of SST trials to that of partial USST as well as full USST trials (see Supplementary Figure S1) showed no significant difference. These results cohesively suggest that the delta activity was associated with sensorimotor information [71]. Accordingly, corresponding targets such as stop signals had similar delta power across conditions. An alternative explanation might be related to working memory load. The Cont_Go signal was used to control for the attentional capture in stop signal trials. Therefore, it may increase the working memory demands of the task [72,73]. On other hand, increased theta power may serve as index of motor inhibition, i.e., there was a larger theta power in NoGo compared to the go trials [38]. In the current study, a larger theta power was observed for SST compared to the Cont_Go trials, from 400 ms to 600 ms following the stop signal. The increased theta band power occurred after the stop signal reaction time, suggesting that theta oscillations cannot entirely account for the inhibitory process, since it may appear too late to be involved in inhibition. Additionally, theta oscillations in SST trials may be associated with the evaluation of the inhibitory process [74].

### 4.2. Neural Mechanisms of Error Detection and Correction

The observation of an increase in theta and alpha band in full USST may be associated with error detection and is consistent with results in previous studies [18,46]. Previously, error monitoring was chiefly linked to mid-frontal theta oscillations that seemingly intensify when an error occurs [19,75,76]. Additionally, increased theta and alpha band after an error is recognized as oscillatory activity that is involved in action monitoring and error detection [18,46]. However, an increase in alpha power has also been associated with top-down attention, which plays a vital role in the sensory detection of inhibition signals [77,78]. We found no changes in the alpha amplitude for SST and Cont_Go conditions in this analysis. This suggests that the Cont_Go signal mimics the attentional capture effect from the stop trials. Therefore, the observation of the increase in the alpha band in full USST may be associated with error detection.

Interestingly, higher alpha and theta band activity was observed in partial versus full USST. This suggests different processes related to error monitoring across conditions (partial USST vs. full USST), such as the alpha power increase being related to particular error types [46,79]. Moreover, higher alpha and theta band activity was observed in partial over both Cont_Go and full USST trials in the window 100 ms before the response onset. A possible explanation for this might be that error detection in partial USST occurred earlier than in full USST to trigger correction of a wrong response. Moreover, since it takes about 20 ms for information to travel from the primary motor cortex to the hand muscles [29], the error detection in partial USST should have occurred before full USST by at least 20 ms. According to the early enhancement of theta and alpha in partial USST, we can infer that humans can detect their errors before or at the very beginning of execution of an erroneous response. Therefore, from this perspective, the larger and earlier theta and alpha in the frontal reflect the early detection of error and correction of the error. 

## 5. Conclusions

In conclusion, the current study used a selective stop signal task and combined it with force degree measurement to explore inhibition and error monitoring mechanisms. Higher beta and low gamma band activity observed for successful stop trials demonstrated that these may serve as a potential electrophysiological index of motor inhibitory control. Additionally, higher theta and alpha band activity in full USST might serve as promising measures of error detection. In contrast, those bands showing activity in partial USST may be associated with early error detection and error correction. These results also suggest different processing related to error monitoring across different types of error. The current study, which aimed to better understand the neural dynamics of motor response inhibition and error monitoring, employed the non-linear Hilbert–Huang transform method for analysis of force measures and electrode-level EEG to allow a novel assessment of cognitive control and, in particular, early error detection and error correction.

## Data Availability

The data that support the findings of this study will be available from the corresponding author upon reasonable request.

## Supplementary Materials

The following are available online at https://www.mdpi.com/article/10.3390/brainsci11040478/s1, Figure S1: Topography of the differences in time-frequency spectrum between SST and full USST (A) as well as for SST and partial USST (B) in the time window from −300 to 600 ms.
