# Supplementary material for: Dynamical EEG Indices of Progressive Motor Inhibition and Error-Monitoring
Source: Brain Sci. 2021 Apr 9;11(4):478. doi: 10.3390/brainsci11040478 (PMC8070019; doi:10.3390/brainsci11040478)
Supplement: Supplementary file 1 [file brainsci-11-00478-s001.pdf]

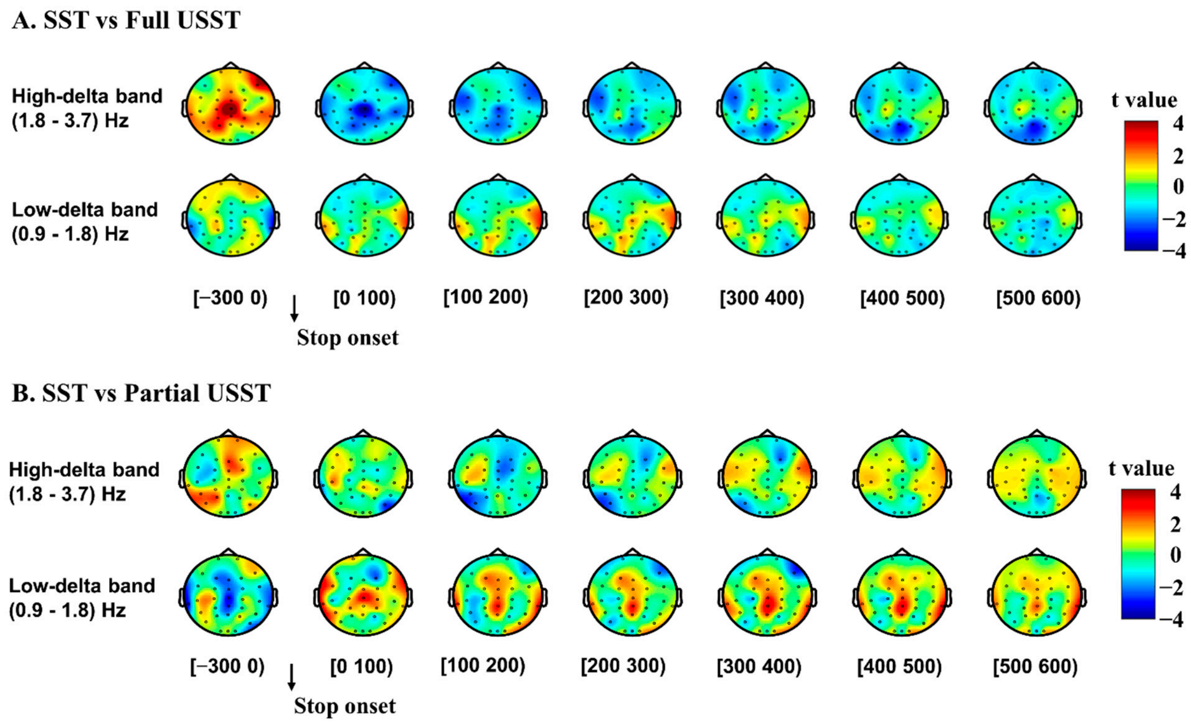

Figure S1: Topography of the differences in time-frequency spectrum between SST and full USST **(A)** as well as for SST and partial USST **(B)** in the time window from -300 to 600 ms. The trial time window was separated into seven time-bins with each topography shown for each time-bin. The time window of the baseline consisted of a single time bin of 300 ms. Data for analysis from all trials were time-locked to the stop onset. There was no significant difference in the delta (low and high-delta) oscillations between SST and full USST conditions (**Figure S1.A**, all $p > 0.05$, CBnPP) as well as between SST and partial USST conditions (**Figure S1.B**, all $p > 0.05$, CBnPP)

Note: the right color bar displays the t value (red denotes positive t values and blue denotes negative t values). White circles around an EEG channel indicates a significant effect for that channel, $p < 0.05$, two-tailed CBnPP test.
